# Oncolytic Virotherapy in Peritoneal Metastasis Gastric Cancer: The Challenges and Achievements

**DOI:** 10.3389/fmolb.2022.835300

**Published:** 2022-02-28

**Authors:** Su Shao, Xue Yang, You-Ni Zhang, Xue-Jun Wang, Ke Li, Ya-Long Zhao, Xiao-Zhou Mou, Pei-Yang Hu

**Affiliations:** ^1^ Department of General Surgery, Chun’an First People’s Hospital (Zhejiang Provincial People’s Hospital Chun’an Branch), Hangzhou, China; ^2^ General Surgery, Cancer Center, Department of Hepatobiliary and Pancreatic Surgery and Minimally Invasive Surgery, Key Laboratory of Tumor Molecular Diagnosis and Individualized Medicine of Zhejiang Province, Zhejiang Provincial People’s Hospital (Affiliated People’s Hospital of Hangzhou Medical College), Hangzhou, China; ^3^ Clinical Research Institute, Zhejiang Provincial People’s Hospital (Affiliated People’s Hospital of Hangzhou Medical College), Hangzhou, China; ^4^ Department of Traumatology, Tiantai People’s Hospital of Zhejiang Province (Tiantai Branch of Zhejiang People’s Hospital), Taizhou, China; ^5^ Guangdong Techpool Bio-pharma Co., Ltd., Guangzhou, China

**Keywords:** oncolytic virotherapy, peritoneal gastric cancer, metastasis, anticancer, immunopathogenesis

## Abstract

Gastric cancer (GC) is the fourth most common cancer and the second leading cause of cancer death globally. Although the mortality rate in some parts of the world, such as East Asia, is still high, new treatments and lifestyle changes have effectively reduced deaths from this type of cancer. One of the main challenges of this type of cancer is its late diagnosis and poor prognosis. GC patients are usually diagnosed in the advanced stages of the disease, which is often associated with peritoneal metastasis (PM) and significantly reduces survival. This type of metastasis in patients with GC poses a serious challenge due to limitations in common therapies such as surgery and tumor resection, as well as failure to respond to systemic chemotherapy. To solve this problem, researchers have used virotherapy such as reovirus-based anticancer therapy in patients with GC along with PM who are resistant to current chemotherapies because this therapeutic approach is able to overcome immune suppression by activating dendritic cells (DCs) and eventually lead to the intrinsic activity of antitumor effector T cells. This review summarizes the immunopathogenesis of peritoneal metastasis of gastric cancer (PMGC) and the details for using virotherapy as an effective anticancer treatment approach, as well as its challenges and opportunities.

## 1 Introduction

Gastric cancer (GC) is considered one of the most common human cancers, and it is the third leading cause of global cancer deaths (Jemal et al., 2011; Olnes and Martinson, 2021). Evidence showed that GC has higher cytologic, genetic, and architectural heterogeneity than other human gastrointestinal malignancies (Abdi et al., 2021). Due to the poor prognosis of GC, it has been shown that this type of cancer has a low 5-year overall survival (OS), which even after treatment with surgery and chemotherapy as well as other therapeutic approaches such as biological treatments, the OS rate in patients according to different continents has been reported between 20 and 60% (Sant et al., 2009; Wei et al., 2016; Olnes and Martinson, 2021). According to the available knowledge, due to the presence of immunosuppressive cells and mediators, as well as the overexpression of inhibitory molecules on the tumor’s surface cells in the tumor microenvironment (TME) of GC, cancerous cells have a strong tendency to invade and metastasize to other organs in the body (Yuki et al., 2020). Among patients with advanced GC, peritoneal implantation is one of the most common and worst metastasis forms. Studies have reported that the peritoneal metastasis (PM) rate of GC patients at the initial phase of the examination was about 14%, and also the median survival time was approximately 3–6 months (Thomassen et al., 2014). Until the early 1990s, PM of GC was considered a terminal disorder due to its unresectability as well as resistance to systemic chemotherapy (Yonemura et al., 2017). Nevertheless, in the late 1990s, conversional therapy was recommended by researchers as a novel therapeutic approach with the aim of *en bloc* resection of macroscopically obvious lesions employing gastrectomy, peritonectomy, and lymphadenectomy, along with the ample removal of peritoneal micrometastasis via perioperative chemotherapy (Yonemura et al., 2017; Wang et al., 2019). However, most clinical studies on peritoneal malignancies are challenged by the continual high rates of peritoneal recurrence and reduced patient survival (Thadi et al., 2018). In this regard, the growing use of novel therapeutic approaches, including immunotherapy-based methods and oncolytic virotherapy in the management of metastatic malignancies, has led to research into translation applications for primary and metastatic peritoneal diseases (Morano et al., 2016). The strength of virotherapy over other therapies is the direct killing of tumor cells without damaging normal and non-tumor cells and tissues, and this advantage clearly emphasized the need to study this treatment (Fukuhara et al., 2016).

Since the early 20th century, there has been speculation that viruses may be used to treat cancer, and some viruses, such as rabies virus, have been studied in the field since the mid-nineteenth century and have shown relatively satisfactory results in tumor regression (Pack, 1950; Southam and Moore, 1952; Sinkovics and Horvath, 1993; Sinkovics and Horvath, 2000). In the following years, the anticancer effects of several other viruses, such as flavivirus West Nile virus (strain Egypt 101), bovine enterovirus, Newcastle disease virus (NDV), oncolytic serotype adenovirus type 4, and the paramyxoviruses mumps, were used in human studies as well as animal models of cancer (Southam and Moore, 1952; Asada, 1974; Okuno et al., 1978). A major challenge in treating patients with peritoneal metastasis of gastric cancer (PMGC) is resistance to chemotherapy which can impair the effectiveness of systemic chemotherapy (Rau et al., 2019). To address this issue, researchers have used reovirus-based anticancer therapy in patients with the chemotherapy-resistant form of PMGC because it can activate dendritic cells (DCs), restore suppressed immune responses and ultimately lead to activation of antitumor CD8^+^ T lymphocytes (Gujar et al., 2010). Experimental and human studies have so far yielded relatively acceptable outcomes from this type of treatment. In this regard, it has been reported that reovirus-based immunotherapy can delay the expansion of PM and increase animal survival via decreasing myeloid-derived suppressor cells (MDSC), regulatory T cells (Tregs), and increasing CD3^+^/CD8^+^ effector T cells and interferon-gamma (IFN-γ) production in studied mice (Gujar et al., 2013). Despite the advantages of virotherapy in the treatment of cancer, similar to other therapeutic approaches, this method is also encountered with relatively similar challenges, including the presence of immunosuppressive TME, lack of proper penetration into the tumor mass, and lack of specific therapeutic therapy biomarkers as well as off-target infections and anti-virus responses immune system.

Therefore, this review aimed to summarize the limitations of PMGC treatment and the reasons for the tendency to use other therapeutic tactics such as virotherapy. Furthermore, the details of the virotherapy are also discussed, along with the challenges facing this type of cancer therapy.

## 2 Peritoneal Metastasis of Gastric Cancer

**FIGURE 1:**
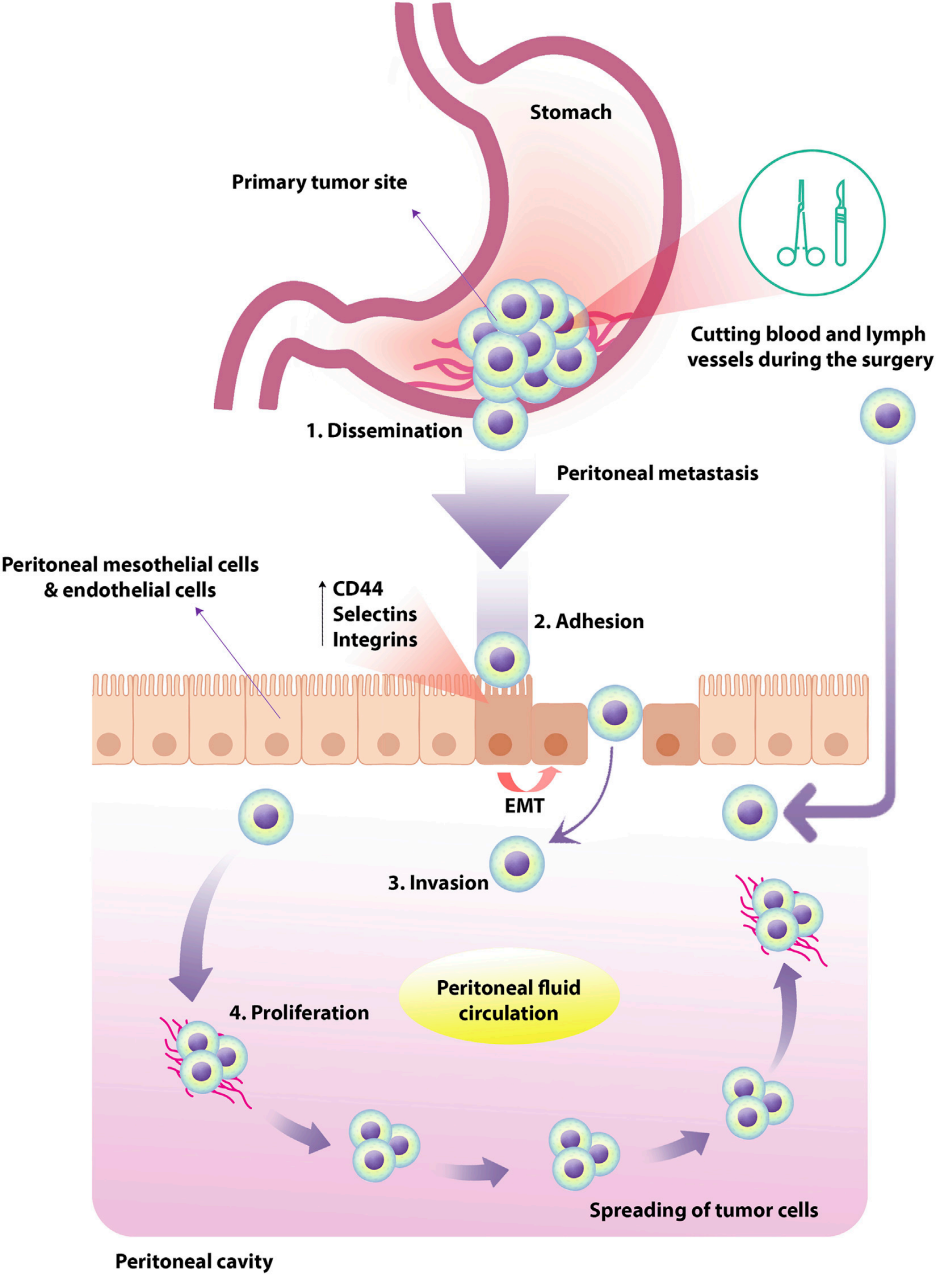
Peritoneal metastasis of tumor cells in human GC. Primary tumor cells originate from the primary abdominal organs and spread through the transcolumic mechanism. The specific type and direction of peritoneal fluid circulation can lead to the tumor cells spreading in a particular order. In human GC, PM occurs in four steps; dissemination, adhesion, invasion, and proliferation. The expression of TGF-β1, leukocyte-associated adhesive molecules such as CD44, selectins and integrins could up-regulate by peritoneal mesothelial cells and endothelial cells, resulting in EMT of peritoneal mesothelial cells. Tumor cells exfoliate from the primary tumor into the peritoneal cavity in the more common transverse growth method, regularly occurring before surgery. In the intraperitoneal spread due to surgical injury, malignant cells are inadvertently released and spread through the peritoneum by manipulating the primary tumor, cutting blood and lymph vessels during the operation. GC, gastric cancer; PM, peritoneal metastasis; TGF-β1, transforming growth factor-beta1; EMT, epithelial-mesenchymal transition.

Evidence showed that PM is one of the most frequent types of metastasis in GC and up to 14% of newly diagnosed GC patients (Kang et al., 2021). Furthermore, the peritoneum is considered the most common site of recurrence upon radical surgery in GC patients (Sugarbaker et al., 2003; Thomassen et al., 2014). It has been reported that in patients with PMGC, due to low treatment efficacy and its challenges, the median survival time of these patients is short and about 3–6 months (Ishizone et al., 2006; Thomassen et al., 2014). However, the cellular and molecular mechanisms underlying PMGC are not yet fully understood. Metastasis of tumor cells as a multistage process is a complex phenomenon. Peritoneal metastasis of GC tumor cells consists of several steps based on available knowledge, including dissemination, adhesion, invasion, and proliferation. Primary malignant cells can migrate to other areas and tissues through the blood, lymph nodes, and local invasion (Bogenrieder and Herlyn, 2003) (Figure 1).

In PM, the primary tumor cells originate from the primary abdominal organs and propagate through the transcolumic mechanism. The specific type and direction of peritoneal fluid circulation can lead to the dispersion of tumor cells in a specific state that depends on multilevel cellular and molecular reactions between peritoneal components and the initial site of malignant cell growth. In this regard, it has been shown that the expression of TGF-β1, leukocyte-associated adhesive molecules such as CD44, selectins and integrins could up-regulate by peritoneal mesothelial cells and endothelial cells, resulting in epithelial-mesenchymal transition (EMT) of peritoneal mesothelial cells (Sun et al., 2017). Following these events, the proliferation of invasive species tumor cells could be increased (Mikuła-Pietrasik et al., 2018). Due to common gastrointestinal cancers, peritoneal carcinomatosis can occur through transversal growth (synchronous peritoneal carcinomatosis) and intraperitoneal spread (*metachronous* peritoneal carcinomatosis). Cancer cells exfoliate from the primary tumor into the peritoneal cavity in the more common transverse growth method, usually occurring before surgery. In the intraperitoneal spread due to surgical injury, malignant cells are inadvertently released and propagated through the peritoneum by manipulating the primary tumor, cutting blood and lymph vessels during the operation (Terzi et al., 2014). Previous studies in this field categorized the spread of peritoneal cancer into three types: Random Proximal Distribution (RPD), Complete Redistribution (CRD), and Wide Cancer Distribution (WCD). Understanding these patterns can greatly affect treatment management and clinical outcomes. It is useful because, for example, the best treatment for RPD is selective peritonectomy of macroscopically involved sections, while for WCD and CRD, complete peritonectomy and cytoreduction treatment are more desirable. Studies demonstrated that among these patterns, RPD occurs in early implantation of moderate and high-grade tumors such as GC in order to the existence of adherence molecules on the cancer cells near the tumor site (Kusamura et al., 2010).

## 3 Peritoneal Metastasis of Gastric Cancer Therapy

Based on available knowledge, systemic chemotherapy is considered the standard cancer therapy method for patients with PMGC (Ishigami et al., 2017). Regarding the outcomes of pivotal clinical trials, the combination of capecitabine or S-1 (Tegafur, Gimeracil, Oteracil) with oxaliplatin or cisplatin is suggested for first-line chemotherapy, and ramucirumab with paclitaxel is also recommended for second-line chemotherapy (Association JGC, 2017). Current improvement in systemic chemotherapy could enhance patients’ prognosis; nonetheless, the median survival time has been extended to only around 1 year (Koizumi et al., 2008; Kang et al., 2009; Wilke et al., 2014; Yamada et al., 2015). Although it has been possible to improve the prognosis of patients with PMGC through chemotherapeutic agents and new molecular targeting, the effectiveness of treatment is still unsatisfactory (Wang et al., 2019). Researchers believe that combination therapy with surgery and chemotherapy can dramatically reduce the size and regression of metastatic tumor lesions and sometimes even the complete disappearance of the tumor (Bang et al., 2010). However, this type of treatment (gastrectomy and postoperative chemotherapy) could not lead to greater efficacy or survival than chemotherapy alone due to the lack of adherence to chemotherapy following surgery (Fujitani et al., 2016). In contrast, other studies aimed at R0 resection (a microscopically margin-negative resection) on cancers that are initially only partially resectable or non-resectable have shown that the use of a multidisciplinary model of conversion therapy through surgical intervention followed by chemotherapy (only in responders to chemotherapy) could be safe and lead to increased survival of patients with PMGC (Ishigami et al., 2017).

## 4 Oncolytic Virotherapy

Virotherapy has been studied for cancer treatment since the 19th century, but due to genetic engineering challenges and concerns about self-immune responses, it has not progressed much in the last 2 decades (Goradel et al., 2021). Genetic engineering aims to modify viral genomes to replicate in cancer cells selectively, and lysis is performed without affecting normal cells. Virotherapy is now considered a form of cancer immunotherapy because oncolytic virus therapy induces immune responses against viral, anti-epitopes in virus-infected tumor cells as well as the death of these tumor cells (Davis and Fang, 2005; De Munck et al., 2017). The United States food and drug administration (FDA) approved T-VEC, a modified form of herpesvirus type 1 (HSV-1), as the first oncolytic virus in 2015 to treat melanoma (Aurelian, 2016). Deleting specific genes in this type of virus can lead to selective proliferation in tumor cells and increase the presentation of tumor and viral antigens to immune effector cells (Pol et al., 2016). Regarding the use of genetic engineering in virotherapy, it has been shown that the gene of cytokines such as the granulocyte-macrophage colony-stimulating factor (GM-CSF) gene promotes the growth factor development and prolongation of cellular and humoral immune responses is inserted in the HSV-1 genome (Rehman et al., 2016). Moreover, in other countries, Oncorine and RIGVIR (enteric cytopathic human orphan type 7) have also been approved as oncolytic viruses for cancer therapy. Oncorine, a genetically modified type 5 human adenovirus (HAdV-C5) in which the E3 and E1B-55KD regions were deleted to stimulate selective virus replication in p53-impaired cells and enhance the safety of the treatment (Goradel et al., 2021). In 2005, China’s state food and drug administration confirmed Oncorine (H101) for head and neck squamous cell carcinoma (Goradel et al., 2021). Furthermore, RIGVIR, a strain from the *Picornaviridae* family, is a no-genetically engineered virus employed to treat melanoma (Doniņa et al., 2015; Alberts et al., 2016). Recent studies show that among the wide range of oncolytic viruses that have been investigated so far, members of the *poxviruses* are the most hopeful candidates for different types of tumors. For example, the oncolytic myxoma virus (MYXV), as a member of the *Leporipoxvirus* genus, contrasting other oncolytic viruses, only infects rabbits and does not harm humans. However, MYXV can selectively infect tumor cells of humans, mice, and some other species, resulting in lysis of these infected tumor cells (Rahman and McFadden, 2020). As mentioned before, among the studied oncolytic viruses, only T-VEC has FDA-approved labeling for use in the treatment of melanoma and investigations on other viruses are underway. Table 1 shows some of the most important completed clinical trials on the use of oncolytic viruses in human malignancies.

**TABLE 1:** Completed clinical trials of oncolytic viruses.

	Virus	Genetic manipulation	Tumor type	Phase	References
*HSV viruses*	G207	None	Brain tumor	II	NCT04482933
	ONCR-177	IL-12, CCL4, FLT3LG, αCTLA4 and αPD-1	Melanoma and other solid tumors	I	NCT04348916
	OH2 (HSV-2)	GM-CSF	Gastrointestinal tumors and other solid tumors	I and II	NCT03866525
	RP1	GALV-GP and GM-CSF	Cutaneous squamous cell carcinoma	Ib	NCT04349436
	RP1	GALV-GP and GM-CSF	Cutaneous squamous cell carcinoma	I	NCT04050436
	RP2	GALV-GP and GM-CSF	Advanced solid tumors	I	NCT03767348
	T-VEC	GM-CSF	Breast Cancer	I	NCT04185311
	T-VEC	GM-CSF	Angiosarcoma of skin	II	NCT03921073
	T-VEC	GM-CSF	Sarcoma	II	NCT03069378
	T-VEC	GM-CSF	Cutaneous melanoma	II	NCT03842943
*Adenoviruses*	CG0070	GM-CSF	Bladder cancer	II	NCT02365818
	Delta-24-RGD	None	Brain tumor	I and II	NCT01582516
	MG1-MAGEA3	MAGEA3	NSCLC	I and II	NCT02879760
	CG0070	GM-CSF	Bladder cancer	II	NCT02365818
*Vaccinia viruses*	Pexa-Vec	GM-CSF	Hepatocellular carcinoma	II	NCT01171651
	Pexa-Vec	GM-CSF	Hepatocellular carcinoma	II	NCT01636284
	Pexa-Vec	GM-CSF	Hepatocellular carcinoma	II	NCT01387555
	GL-ONC1	Luc-GFP	Head and neck cancer	I	NCT01584284
		β-Galactosidase			
		β-glucuronidase			
	GL-ONC1	Luc-GFP	Solid tumors	I	NCT00794131
		β-Galactosidase			
		β-glucuronidase			
	vvDD	Cytosine deaminase and somatostatin receptor	Solid tumors	I	NCT00574977

### 4.1 Oncolytic Viruses Mechanisms of Action

**FIGURE 2:**
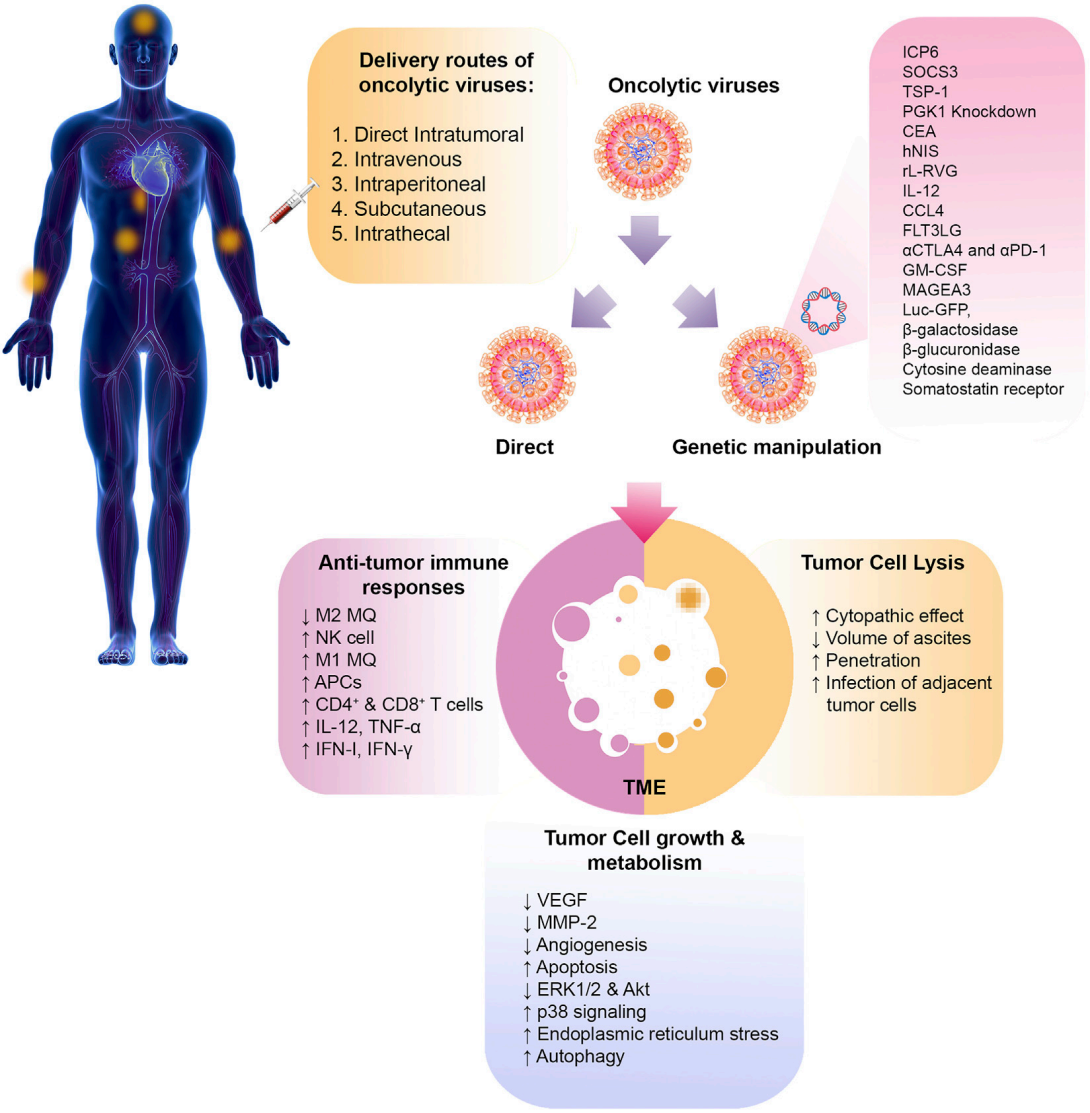
Oncolytic virotherapy of cancer. The various routes of oncolytic virus delivery are shown. Oncolytic viruses can be manipulated through genetic engineering to express specific genes or administered directly without modification. Once they reach the tumor site, these viruses can directly cause lysis of tumor cells. They can also help remove the tumor by altering the immunosuppressive TME and inducingantitumor effector immune cells. Other mechanisms, such as angiogenesis, apoptosis, and autophagy, can also be altered by oncolytic viruses. PMGC, peritoneal metastasis of gastric cancer; SOCS3, suppressor of cytokine signaling 3; TSP-1, thrombospondin-1; PGK-1, phosphoglycerate kinase-1; CEA, carcinoembryonic antigen; hNIS, human sodium iodide symporter; rL-RVG, rabies virus glycoprotein; HSV, herpesvirus; 5-FU, 5-fluoro-uracil; PTX, paclitaxel; NDV, Newcastle disease virus; MQ, macrophage; VEGF, vascular growth factor; MMP-2, matrix metalloproteinase-2; FLT3LG, Fms related receptor tyrosine kinase 3 ligand; CTLA4, cytotoxic T-lymphocyte-associated protein 4; PD-1, programmed cell death-1; GM-CSF, granulocyte-macrophage colony-stimulating factor; MAGEA3, melanoma-associated antigen 3; Luc GFP, luciferase green fluorescent protein; IFN, interferon; TNF, tumor necrosis factor; APC, antigen presenting cell.

Studies have shown that oncolytic viruses can kill cancer through the two primary mechanisms of direct cell lysis and the induction of antitumor immune responses (Figure 2).

#### 4.1.1 Tumor Cell Lysis

Virus replication in infected tumor cells leads to apoptosis in the cell lysis mechanism. Following virus replication in tumor cells and cell lysis, viral particles repeat the lytic cycle by infecting adjacent cancer cells, inducing and amplifying treatment at the target tumor site (Mullen and Tanabe, 2002). The viral lytic cycle continues until infected host cells are depleted, or antiviral immune responses attenuate virus replication (Hamid et al., 2017). Immune responses can also lead to the death of tumor cells by breaking the tolerance of tumor cells (Workenhe et al., 2015; van Vloten et al., 2018). Non-infectious host cells can also be affected by oncolytic viruses in favor of treatment. In this context, it has been disclosed that the oncolytic vaccine virus can interrupt tumor angiogenesis, reduce blood flow to cancer cells, and ultimately cause hypoxia by affecting vascular cells, all of which are associated with inhibiting tumor growth and progression (Breitbach et al., 2007; Breitbach et al., 2013; Hashemi Goradel et al., 2018). Although lysis of tumor cells through the initiation of the lytic cycle is one of the inherent characteristics of oncolytic viruses, evidence suggests that further manipulations can increase their lytic capacity. For instance, the herpes simplex virus-1 thymidine kinase (HSV-1 TK) expresses adenovirus (Ad-OC-HSV-TK), in which the expression of HSV-1 TK is under the osteocalcin promoter, to target tumor cells in designed for bone malignancies (Kubo et al., 2003; Goradel et al., 2021). In this regard, HSV-1 TK can activate thymidine analogs such as ganciclovir as a competitive inhibitor of deoxyguanosine by conversion to monophosphates. Monophosphates can also disrupt and terminate DNA synthesis by inserting proliferating cells DNA, resulting in cell death (Alvarez and Curiel, 1997). Another suicidal gene under study is cytosine deaminase (CD), which can convert 5-fluorocytosine to 5-fluorouracil with high cytotoxic properties (Freytag et al., 2002). The insertion of the ADP gene into the adenovirus genome upsurges the lytic activity of the virus. ADP is also involved in encoding the adenovirus death protein (ADP), which is crucial for the infection of type C adenoviruses in the later phases of infection and the spread of viral particles (Doronin et al., 2000).

#### 4.1.2 Enhancement of Anti-Tumor Immune Responses

The second mechanism of action oncolytic viruses is to increase antitumor immune responses. Studies have shown that following infection of tumor cells with oncolytic viruses, cell death and the release of tumor-related antigens such as viral pathogen-associated molecular patterns (PAMPs) and different cellular danger-associated molecular patterns (DAMPs) lead to the enhancement of tumor-specific immune responses and the killing of distant and non-infectious tumor cells (Pol et al., 2012). Tumor cell lysis can also induce the production and secretion of inflammatory mediators, including type I interferons (IFNs), interferon-gamma (IFN-γ), interleukin-12 (IL-12), and tumor necrosis factor-α (TNF-α) (Kaufman et al., 2015). The philosophy of using engineered oncolytic viruses is to enhance immune responses further. In this strategy, the insertion of an immune-stimulating molecule into the oncological genome of viruses could alter the immune-suppressive tumor microenvironment in favor of treatment. As previously mentioned, GM-CSF is the most obvious example of this type of genetic engineering. After incorporating the GM-CSF gene into the oncolytic genome, viruses can act as an immune responses stimulator, leading to the maturation and recruitment of antigen-presenting cells (APCs), particularly DCs, inducing antitumor effector T cells and NK cells which are specific for tumor antigens (Jhawar et al., 2017). In order to improve and increase the delivery of intracellular antigen to the proteasome and antigen presentation, the oncolytic adenovirus genome was modified for overexpression of heat shock proteins (Hsp70) protein, and the outcomes disclosed that the frequency of CD4^+^ and CD8^+^ T cells along with NK cells increased following the administration of this type of modified oncolytic adenovirus (Li et al., 2009). Correspondingly, due to the expression of Hsp receptors such as CD91 (α2-macroglobulin receptor or the low-density lipoprotein–related protein) and lectin-like oxidized low-density lipoprotein receptor-1 (LOX-1), HSP70 in APCs, the delivery of tumor antigen to APCs is improved through this approach (Nishikawa et al., 2008).

### 4.2 Oncolytic Viruses Used in Cancer Therapy

Numerous oncolytic viruses have been used to treat malignancies. Among these viruses, adenoviruses, HSVs, vaccinia virus, Newcastle disease virus (NDV), coxsackievirus, measles virus (MeV), Seneca Valley virus, poliovirus, parvovirus, vesicular stomatitis virus, and the Maraba virus are the most investigated in cancer therapy (Davis and Fang, 2005).

### 4.3 Delivery Routes of Oncolytic Viruses

Studies on cancer treatment using oncolytic viruses have shown that non-optimal delivery is one of the main reasons for treatment failure. Several delivery routes for oncolytic virus therapy have been investigated, and their proper selection based on research objectives is essential to increase the effectiveness of treatment (Figure 2). This section briefly introduces common oncolytic viruses delivery methods in cancer therapy.

#### 4.3.1 Direct Intratumoral Delivery

Direct intratumoral delivery is the most common route of administration of oncolytic virus in patients with cancer. In this method, the concentration of oncolytic virus in the desired site can be accurately managed and controlled, and on the other hand, the adverse effects caused by the improper transmission of the virus to other organs can be prohibited. According to the obtained outcomes, due to operational complications in direct intratumoral delivery, it is much more suitable for superficial tumors such as melanoma than deep tumors such as glioblastoma (Li et al., 2020).

#### 4.3.2 Intravenous Delivery

Intravenous delivery of oncolytic viruses is a simple administration route for physicians in cancer therapy. Numerous researchers in clinical trials using oncolytic viruses prefer intravenous injections to intratumoral injections because they believe that intratumoral injections have several challenges and complexities, such as surgery for deep-seated tumors as well as delivery barriers in high metastatic malignancies (Waters et al., 2017; Komorowski et al., 2018; Samson et al., 2018; Tang et al., 2019). It has been shown in several human malignancies that intravenous injection of oncolytic viruses can induce tumor elimination through various mechanisms such as alteration of the immunosuppressive TME by reducing the expression of inhibitory molecules as well as affecting immune cells by increasing their antitumor function (Waters et al., 2017; Komorowski et al., 2018; Samson et al., 2018; Tang et al., 2019). Intravenous delivery of oncolytic viruses can also facilitate the passage of various barriers such as the extracellular matrix (ECM) and blood-brain barrier (BBB), which are the main challenge in the transmission of the oncolytic virus in solid tumors (Choi et al., 2012). However, the immune clearance of oncolytic viruses and insufficient concentration of viruses reaching the tumor site can disadvantage intravenous delivery.

#### 4.3.3 Intraperitoneal Delivery

Because the peritoneal cavity is a large area, absorption of intraperitoneally injectable drugs and compounds is faster than drug administration via subcutaneous injection. While drug absorption by intraperitoneal injection is slower than intravenously injected drugs. Another advantage of intraperitoneal administration is the relative ease of injection, which does not require any specialized skills. It appears that if the organs inside the abdominal cavity are the target of treatment, intraperitoneal injection is an ideal and smart choice for the delivery of oncolytic viruses (Li et al., 2020; Chen et al., 2017; O’Leary et al., 2018).

#### 4.3.4 Subcutaneous and Intrathecal Delivery

Subcutaneous injection is also a fairly common method of administering oncolytic viruses. This method is particularly used for small animals whose veins are hard to find (Kuryk et al., 2017). Additionally, the possibility of intrathecal injection is limited to the central nervous system (CNS)-related tumors. By way of explanation, due to the low efficiency of subcutaneous and intrathecal delivery approaches, these methods are less used and are principally limited to animal experiments (Ochiai et al., 2006).

## 5 Oncolytic Virotherapy in Treatment of Peritoneal Metastasis of Gastric Cancer

As mentioned earlier, the prognosis of patients with PMGC is very poor, and related investigations are needed to find an effective treatment given the limitations and shortcomings of previous routine treatments such as surgery and chemotherapy. Few studies have been performed to evaluate the efficacy of oncolytic viruses in the treatment of PMGC.

### 5.1 *In Vitro* Studies

An investigation on GC cell lines including SGC-7901 and AGS infected with the NDV wild-type strain and the recombinant avirulent NDV LaSota strain expressing the rabies virus glycoprotein (rL-RVG) showed that the growth of studied cells in the rL-RVG-infected group was significantly inhibited compared with the wild-type NDV-infected group. RL-RVG and NDV also increase endoplasmic reticulum stress, autophagy, and apoptosis in SGC-7901 and AGS cells. Immunofluorescence analysis in this study disclosed that the mitochondrial membrane was collapsed. It has been revealed that beclin-1 participated in the Bcl-2/Bcl-xL complex activity and inhibition of the formation of the autophagosomes (Cho et al., 2009). In this context, the findings showed that the expression of beclin-1 increased in virus-infected cells, reducing the beclin-1 and Bcl-2/Bcl-xL interaction as well as inducing apoptosis and autophagy. These outcomes collectively suggested that NDV and rL-RVG could induce stomach adenocarcinoma cell death via apoptosis and autophagy along with dysfunction of the endoplasmic reticulum and mitochondria (Bu et al., 2015).

Based on previous studies, phosphoglycerate kinase 1 (PGK1) can participate in PMGC and impact the tumor stem cell’s growth and differentiation in GC (Zieker et al., 2008; Zieker et al., 2013). A study by hairpin RNA knockdown of PGK1 through adenovirus-shPGK-1 and using the chemotherapeutic agents 5-fluoro-uracil (5-FU) and mitomycin showed that mitomycin and 5-FU alone could significantly reduce tumor cells viability. This study also showed that treatment with AdvshPGK-1 alone has an improved effect on reducing tumor cell viability. To determine the effect of combination therapy, 5-FU and mitomycin were used simultaneously with adenovirus-shPGK-1, and the outcomes disclosed that this treatment could be more effective than using either 5-FU, mitomycin or AdvshPGK-1 alone. These findings indicate that inhibition of PGK-1 can increase the susceptibility of metastatic GC cells and tumor stem cells to overcome the chemotherapeutic therapy resistance (Schneider et al., 2015).

### 5.2 *In Vivo* Animal Model Studies

A study was performed using an experimental PMGC animal model to use serotype three oncolytic reoviruses to treat PM in human GC by evaluating the cytopathic effect of reovirus and activity of Ras in human GC cell lines *in vitro*. After reovirus infection, the cytopathic effect was reported in GC cell lines without affecting normal control cells. The Ras activation assay showed Ras’s activity increased in all GC cell lines (MKN45p, NUGC4, MKN7) compared to control cells (KatoIII). Correspondingly, the animal model of PMGC using systemic delivery of reovirus showed that the mean number of tumor cells and weight of total peritoneal tumors along with the volume of ascites were significantly reduced in the treated group compared to the control group. The outcomes of this study indicate that intraperitoneal administration of reovirus might be useful as a novel treatment in PMGC (Kawaguchi et al., 2010).

It has been revealed that to inhibit the growth of human epidermal growth factor receptor 2 (HER2)-overexpressing GC cells, using trastuzumab (anti-HER2 receptor mononuclear antibody) could be effective. The question arises as to whether combination therapy employing oncolytic reovirus and trastuzumab could offer a novel and more effective treatment option for GC. A mouse GC xenograft transplantation model study explored the therapeutic impacts of oncolytic reovirus and trastuzumab to answer this question. Molecular analysis of pathways associated with cell damage was measured by PCR array, and the expression of proteins involved in cell proliferation and apoptosis was examined by western blotting. The results showed that reovirus could sensitize GC cells by overexpressing HER2 for apoptosis. The outcomes of *in vitro* and *in vivo* experiments provided evidence that the combination of oncolytic reovirus and trastuzumab is a more effective method against HER2-overexpressing GC cells than using reovirus or trastuzumab alone. Molecular analysis showed that oncolytic reovirus and trastuzumab could induce higher tumor necrosis factor-related apoptosis-inducing ligand or Apo 2 ligand (TRAIL/Apo2L) in cancer cells.

Moreover, in this study, antibodies against TRAIL strongly reduced combination therapy-associated cytotoxicity. These findings suggested that reovirus might upsurge trastuzumab-induced cytotoxicity in GC cells (Hamano et al., 2015). It appears that upon the combination therapy, released TRAIL from tumor cells might stimulate antitumor responses such as anti-angiogenic responses and antibody-dependent cellular cytotoxicity (ADCC) in an autocrine manner; because according to the findings of this study, tumor xenografts in the nude mice only eradicated in reovirus and trastuzumab treated group.

The employment of G47Δ, the third generation of oncolytic HSV-1, is considered a novel and attractive therapeutic approach for solid tumors. In this regard, a study examined the therapeutic potential of G47Δ for human GC, and the results showed that *in vitro* administration of G47Δ showed a satisfactory proliferative and cytopathic impact on several studied human GC cell lines. Moreover, intratumor injection of G47Δ was also able to significantly inhibit the growth of subcutaneous tumors by increasing the expression of immunostimulatory molecules (soluble CD80) and IL-12 and enhancing M1 macrophages polarization and infiltration *in vivo*. Furthermore, the frequency of cytotoxic NK cells increased following G47Δ administration (Sugawara et al., 2020). Studies on orthopedic tumor models and peritoneal diffusion models of GC disclosed that intratumoral or intraperitoneal administration of G47Δ could alter the immunosuppressive TME and its components, including Tregs, MDSCs, and TAMs resulting in more effective trafficking of effector immune cells in tumor site and further antitumor responses (Saha et al., 2017).

On the other hand, the mentioned effector immune cells can induce innate immune antiviral responses and reduce the effectiveness of virotherapy (Fulci et al., 2007; Alvarez-Breckenridge et al., 2012). It has been reported that HSV-induced M1 macrophages can participate in removing virus-infected cells by producing TNF-α (Meisen et al., 2015). However, another study reported that stimulated macrophages by oncolytic viruses that have infiltrated tumor tissue did not lead to virus clearance and had no significant effect on the effectiveness of virotherapy in cancers (Zemp et al., 2014). Since the immune system’s behavior against different viruses is different and the mentioned study was performed on oncolytic myxoma virus in glioma, this finding cannot be generalized to all cancers and oncolytic viruses. Therefore, eliminating the clearance of the virus by the immune system can be of particular importance in the success or failure of cancer virotherapy and further studies are needed in this area. Another study used a telomerase-specific oncolytic adenovirus expressing TRAIL (Ad/TRAIL-E1) to express both the adenovirus early region 1A (*E1A*) and *TRAIL* genes under the control of a specific tumor promoter. The antitumor effect of Ad/TRAIL-E1 on GC cells was evaluated *in vitro* and *in vivo* in a xenograft model of peritoneal carcinomatosis. This investigation demonstrated that Ad/TRAIL-E1 induces TRAIL-mediated apoptosis in GC cell lines and has no effect on normal cell lines, which is beneficial for treatment. In addition, Ad/TRAIL-E1 was able to significantly inhibit PM and increase the survival of mice without long-term toxicity associated with treatment. Thus, tumor-specific TRAIL expressing adenovirus may offer a novel therapeutic approach to treating PMGC (Zhou et al., 2017).

Studies have shown that the low-pathogenic human enterovirus Echovirus 1 (EV1), an oncolytic virus, can selectively target and kill malignant ovarian and prostate cancer cells in xenograft models (Melnick and Ågren, 1952; Shafren et al., 2005; Berry et al., 2008). EV1 infection and the initiation of the lytic cycle in the target tumor cell require the surface expression of the α2β1, a type of integrin that disseminates GC cells into the peritoneum (Koike et al., 1997; Kawamura et al., 2001). Flow cytometry-based analyses have shown that α2β1 integrin is highly expressed on several GC cell lines, making these cells more susceptible to EV1 lytic infection *in vitro* and leading to effective PMGC treatment. One of the animal models used for non-invasive monitoring of tumor burden in the peritoneum is the MKN-45-Luc SCID bioluminescence mice model, which can also be used to determine therapeutic dose-response (Haley et al., 2009). In this model, it has been reported that oncolytic EV1 could be effectively employed to control PMGC. Pre-existing immunity to EV1, such as antiviral neutralizing antibodies, could be a potential barrier in virotherapy. Although preliminary investigations have revealed that the prevalence of anti-EV1 neutralizing antibodies in the population is low (about 6%), this study is relatively old and more studies are needed on different populations to determine the precise prevalence of anti-EV1 neutralizing antibodies (Karttunen et al., 2003).

Thrombospondin-1 (TSP-1), an endogenous anti-angiogenic factor, is able to suppress tumor growth and progression through various mechanisms, such as inhibition of angiogenic pathways (Weinstat-Saslow et al., 1994; Sheibani and Frazier, 1995; Volpert et al., 1997). One approach to enhance the effects of oncolytic HSV is to produce an oncolytic HSV expressing TSP-1, which in addition to oncolysis of tumor cells, can induce anti-angiogenic mechanisms. In the treatment of human GC, a third-generation oncological HSV (T-TSP-1) expressing human TSP-1 was studied *in vitro* and *in vivo*, and the results demonstrated that TSP-1-mediated apoptosis was more inhibited in MKN1 than TMK-1 GC cell *in vitro*. Arming the viruses with TSP-1 had little effect on their proliferation in some GC cell lines but did not reduce their viral cytolysis and antitumor effects. Furthermore, *in vivo* administration of T-TSP-1 in addition to oncolysis could inhibit angiogenesis through suppression of TGF-β signaling (Tsuji et al., 2013). As discussed before, PGK-1 is likely involved in the metastatic spread of tumor cells in GC (Warburg et al., 1927). In addition, PGK-1 has a real effect on tumor stem cell characteristics. The presence of malignant stem cells is significant in therapeutic resistance and recurrence. It is hypothesized that targeting and inhibiting PGK-1 makes these cells more sensitive to chemotherapy, and thus therapeutic resistance can be overcome. A phase III clinical trial study reported promising results using intraperitoneal paclitaxel (PTX) for PMGC (Takashima et al., 2019). However, this treatment has not been effective enough to eradicate PMGC. Whether intraperitoneal oncolytic virus therapy with PTX could be effective in PMGC was investigated by a research team. OBP-401, an attenuated oncolytic adenovirus that can express green fluorescence protein (GFP) driven by the telomerase promoter, was employed in this study and the effect of its combination therapy with PTX on different human GC cell lines (GCIY and KATO III) and xenograft PM model was also evaluated. The results showed that OBP-401 in combination with PTX synergistically reduced the viability of human GC cells and increased the proliferative ability of the virus in cancer cells. This combination therapy also induced mitotic catastrophe, accelerated autophagy, and apoptosis. Administration of PTX in the human orthopedic PMGC model was also able to profoundly increase the penetration of OBP-401 into the disseminated nodules. In this study, a non-invasive *in vivo* imaging system (IVIS) was used, and the imaging results showed that combination treatment of OBP-401 with PTX significantly inhibited the growth of the metastatic peritoneal tumor reduced the volume of malignant ascites. Although based on these findings, intraperitoneal virus therapy with PTX is considered a promising treatment approach for PMGC; clinical trials are necessary to evaluate the effectiveness of this type of combination therapy in patients with PMGC (Ogawa et al., 2019).

Although adenoviral gene therapy has been described as a potentially promising therapeutic approach, dose-limiting toxicity and reported in clinical trials adverse effects, including flu-like symptoms, transaminitis and lymphopenia, are considered challenges of using adenovirus vectors (Lan et al., 1997; Heise et al., 1999; Reid et al., 2002). To solve this problem, a new system using adenoviral oncolytic suicide gene therapy targeting carcinoembryonic antigen (CEA) was constructed, and its beneficial effect and the possibility to decrease the total viral dose by preserving the antitumor effect were evaluated. Three types of adenoviruses were employed for this system: (I) Ad/CEA-Cre, (II) Ad/lox-CD::UPRT for a Cre/loxP system, and (III) Ad/CEA-E1 for persisting adenovirus replication. Then, the antitumor consequence of the oncolytic suicide gene therapy (I + II + III) was assessed *in vitro*. At the same viral dose, the present system (I + II + III) showed pointedly improved cytotoxic impacts for CEA-producing cell lines compared to suicide gene therapy (I + II) *in vitro*. Therefore, it is possible to decrease the total adenoviral dose along whit preserving the antitumor properties of the virus in oncolytic suicide gene therapy (Imamura et al., 2010).

It has been demonstrated that NDV-D90, as an oncolytic virus in Newcastle disease, could induce cell apoptosis in GC tumor cells in a dose-dependent manner in GC cell lines, including BGC-823, SGC-7901 but not in MKN-28 cells MKN-28 (Sui et al., 2017). Additionally, cell invasion was significantly reduced only in BGC-823 and SGC-7901 cells following this type of virus therapy. The decrease in cell growth and the increase in cell apoptosis in GC cells treated with NDV-D90 are probably due to the suppression of ERK1/2 and Akt signaling and the increase of p38 signaling. Moreover, orthotopic injection of NDV-D90 impaired tumor cells implantation and inhibited tumor growth with intra-tumor necrosis *in vivo*. In addition, it appears that NDV-D90 could suppress angiogenesis of gastric tissue by inhibition of vascular endothelial growth factor (VEGF)-A and matrix metalloproteinase-2 (MMP-2), all of which may prevent tumor progress and metastasis (Sui et al., 2017). Since this study explored the effects of NDV-D90 on human GC cells, the TME was in mice. Moreover, the immunodeficiency condition of nude mice may affect the data interpretation.

Based on previous studies, vaccinia-based virotherapy has had hopeful therapeutic impacts on various human cancers with proper safety (Chen et al., 2009). The therapeutic efficacy of a novel genetically-engineered vaccinia virus expressing the human sodium iodide symporter (*h*NIS) gene was investigated, and the outcomes showed that treatment of tumor cells by GLV-1 h153 could efficiently regress GC and permit deep-tissue imaging (Jun et al., 2014).

### 5.3 *In Vitro*/*Ex Vivo* Studies

As previously discussed, oncolytic virus therapy using HSV has emerged as a new therapeutic approach in treating human malignancies (Fukuhara et al., 2016). Evidence shows that telomerase is activated in many malignant tumors, including GC, and that human telomerase reverse transcriptase (hTERT) is one of the key components of the telomerase enzyme (Liu et al., 2012; Yano et al., 2017). Therefore, it can be clinched that the insertion of essential genes under the regulation of the hTERT promoter, such as the ICP6 in oncolytic HSV, may potentiate its antitumor effects. A study of fourth-generation oncolytic HSVs containing the ICP6 gene regulated by the hTERT promoter (T hTERT) showed that this type of virus could have enhanced cytotoxicity in MKN45, MKN28, and MKN1 cells *in vitro* compared to third-generation oncolytic HSV which the mentioned cytotoxicity of T hTERT especially was higher in MKN45 cells. In addition, *ex vivo* assessment of oncolytic HSV cytotoxicity in GC disclosed that a significant percentage of initial clinical tumors were lysed after infection with T null or T hTERT viruses. These findings suggest that the use of oncolytic HSVs containing the *ICP6* gene under the regulation of the hTERT promoter may be a beneficial and effective therapeutic approach for GC (Kato et al., 2021). Recently, another study examined the efficacy of a third-generation HSV oncolytic suppressor of cytokine signaling 3 (SOCS3). Intensification of viral replication and oncolysis of T-SOCS3 for different human GC cell lines was investigated *in vitro*, and the results showed that T-SOCS3 could increase its proliferation and its tumor cell lysis properties for the MKN1 cell line. T-SOCS3 also induces the destruction of tumor cells in human GC specimens (Matsumura et al., 2021).

Taken together, the studies and their results show that viral therapy using different types of oncolytic viruses and also amplifying them by arming these viruses with different genes with antitumor activity may be effective to treat PMGC via various mechanisms such as direct oncolysis, inhibition of angiogenesis and induction of apoptotic as well as autophagic pathways (Table 2).

**TABLE 2:** Oncolytic viruses used in the treatment of PMGC.

Oncolytic virus	Study details	Genetic manipulation	Route	Outcomes	Reference
NDV	*In vitro*	rL-RVG	-	Increasing endoplasmic reticulum stress, autophagy, and apoptosis	Bu et al. (2015)
	• Human GC cell lines: SGC-7901 and AGS				
	• rL-RVG				
Adenovirus +5-FU and mitomycin	*In vitro*	Knockdown of PGK1	-	Reducing tumor cell viability, increasing the susceptibility of metastatic GC cells and tumor stem cells to overcome the chemotherapeutic therapy resistance	Schneider et al. (2015)
	• Human GC cell line: 23132/87 (ACC409)				
	• Adv-shPGK1				
Reovirus	*In vitro*/Animal model	None	IV/IP	Increase of cytopathic effect, increase of Ras activity, Reduce the mean number and weight of total peritoneal tumors along with the volume of ascites	Kawaguchi et al. (2010)
	• Human GC cell lines: MKN45p, NUGC4, MKN7				
	• Reovirus serotype 3				
	• Nude mice				
Reovirus + trastuzumab	*In vitro*/Animal model	None	SQ	Inhibition of HER2, sensitization of GC cells by overexpressing HER2 for apoptosis by reovirus, increase of TRAIL/Apo2L-mediated apoptosis, increasing anti-angiogenic responses and ADCC	Hamano et al. (2015)
	• Human GC cell lines: NCI-N87 & MKN-28				
	• Reovirus serotype 3				
	• Male BALB/c nude mice				
HSV-1 (G47Δ)	*In vitro*/Animal model	None	IT/IP	Satisfactory proliferative and cytopathic effects, decreasing M2 macrophages and increasing M1 macrophages along with NK cells	Sugawara et al. (2020)
	• Human GC cell lines: MKN45, MKN74, and 44As3				
	• G47Δ				
	• Female athymic mice				
Adenovirus	*In vitro*/Animal model	E1A and TRAIL	IP	Antitumor effects, inhibit PM and lead to increase survival	Zhou et al. (2017)
	• Human GC cell lines: MKN45, HGC27, SGC-7901, MKN28, NHFB				
	• Ad/TRAIL-E1				
	• BALB/c nude mice				
Echovirus 1	*In vitro*/Animal model	None	IP	Antitumor effects, oncolysis of α2β1expressing tumor cells	Haley et al. (2009)
	• Human GC cell lines: AGS, Hs746T, and NCI-N87				
	• MKN-45-Luc cells				
	• (SCID)- BALB/c mice				
HSV	*In vitro*/Animal model	TSP-1	SQ	Proliferative and cytopathic effects, Oncolysis of tumor cells, anti-angiogenic effects via inhibiting TGF-β signaling	Tsuji et al. (2013)
	• Vero (Africa green monkey kidney), AZ521, MKN1, MKN28, MKN45 and MKN74 (human GC cell lines)				
	• T-TSP-1 female BALB/c nu/nu mice				
Adenovirus+ PTX	*In vitro*/Animal model	None	IP	Reducing the viability of human GC cells and increasing the proliferative ability of the virus in tumor cells, induction of mitotic catastrophe, accelerated autophagy, and apoptosis, inhibiting the growth of the metastatic peritoneal tumor and reducing the volume of malignant ascites	Ogawa et al. (2019)
	• Human GC cell lines: GCIY and KATO III				
	• OBP-401				
	Xenograft peritoneal metastasis model				
Adenovirus	*In vitro*/Animal model	CEA	IP	Decreasing the total viral dose, preserving the antitumor effect	Imamura et al. (2010)
	• Human GC cell lines: AGS, MKN1, MKN45				
	• Ad/CEA-Cre, Ad/lox-CD::UPRT, and Ad/CEA-E1				
	• BALB/c nu/nu mice				
NDV	*In vitro*/Animal model	None	IT	Inducing cell apoptosis in GC tumor cells, reducing tumor cell invasion, suppression of ERK1/2 and Akt signaling, anti-angiogenic effects by inhibition of VEGF-A and MMP-2	Sui et al. (2017)
	• Human GC cell lines: BGC-823, SGC-7901 and MKN-28				
	• NDV-D90				
	• Male nude mice				
Vaccinia	*In vitro*/Animal model	*h*NIS	SQ	Efficiently regress GC and permit deep-tissue imaging	Jun et al. (2014)
	• Human GC cell lines: AGS, OCUM-2MD3, MKN-45, MKN-74 and TMK-1				
	• GLV-1 h153				
	Female nude mice				
4th-generation oncolytic HSV	*In vitro*/*ex vivo*	ICP6	-	Antitumor effects, oncolysis of tumor cells	Kato et al. (2021)
	• Vero (African green monkey kidney normal cell line), MKN1, MKN28, MKN45, MKN74, NUGC3, NUGC4, KATOIII, and N87 (human GC cell lines)				
	• T-hTERT				
	• Human gastric adenocarcinoma specimens				
3rd-generation HSV	*In vitro*/*ex vivo*	SOCS-3	-	Satisfactory proliferative and cytopathic effects	Matsumura et al. (2021)
	• Human GC cell lines: MKN1, MKN28 and MKN74 cells				
	• T-01				

IP, intraperitoneal; IT, intratumoral; SQ, subcutaneous; IV, intravenous.

## 6 What Are Remaining Challenges?

**FIGURE 3:**
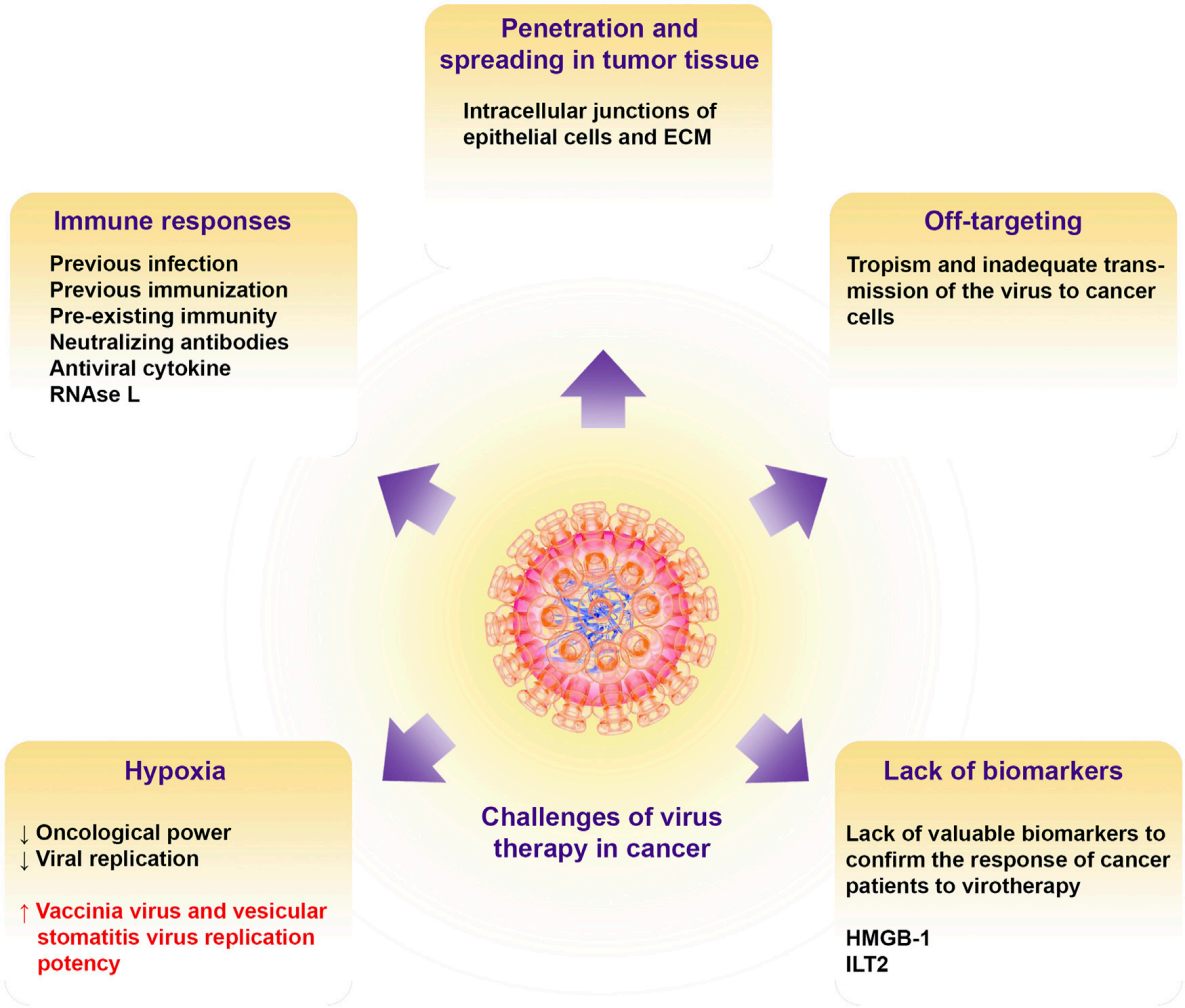
Challenges of oncolytic virotherapy in cancer. Illustrate the barriers to oncolytic viral therapy including tissue penetration, off targeting, immune responses, hypoxic condition in the TME, and lack of putative biomarkers for patient virotherapy monitoring. ECM, extracellular matrix; HMGB-1, high mobility group box-1; ILT2, Ig-like transcript 2.

In this section, the challenges of virus therapy in the treatment of human cancers are discussed and also suggestions for removing these barriers and limitations to increase the effectiveness of treatment are presented (Figure 3).

### 6.1 Oncolytic Virus Penetration and Spreading in Tumor Tissue

Evidence has shown that intracellular junctions of epithelial cells and ECM in carcinomas prevent the penetration of therapeutic agents such as oncolytic viruses, especially adenoviruses, which leads to resistance to treatment and failure of cancer therapy (Lipinski et al., 1997; Green et al., 2002; Christiansen and Rajasekaran, 2006; Lavin et al., 2007). In addition, during metastasis, phenotype alteration through epithelial-to-mesenchymal transition (EMT) and then mesenchymal-to-epithelial transition (MET) makes epithelial junctions tighten, which this event is not in favor of effective treatment (Christiansen and Rajasekaran, 2006; Turley et al., 2008). Some types of adenoviruses, such as B14p, B14, and HAdV-B3, may overcome epithelial junctions by releasing Pantone-dodecahedron (Pt-Dd) in the early phases of infection and before oncolysis. Non-Pt-Dd adenoviruses such as HAdV-C5, which is most commonly used in the production of oncolytic viruses, begin to overproduce fiber protein in the mentioned phase of infection (Fender et al., 2005; Lu et al., 2013). For improved access to cancer cells and oncolysis, navigating the ECM barriers is necessary for oncolytic viruses (Wojton and Kaur, 2010). For this purpose, pretreatment of the tumor cells with collagenase or concomitant administration of hyaluronidase with oncolytic adenoviruses led to more spreading of the virus (Kuriyama et al., 2000; Ganesh et al., 2008). In order to increase therapeutic efficacy, the engineering of oncolytic viruses for the expression of MMP-1 and MMP-8 leads to the degradation of tumor-associated sulfated glycosaminoglycans, which increases virus penetration and dissemination (Mok et al., 2007). Induction of apoptosis by cytotoxic agents and activation of caspase-8 has been reported to increase intra-tumor infiltration and thus antitumor efficacy of oncolytic HSV. It has been interpreted that shrinkage or initiation of apoptotic pathways in tumor cells leads to the formation of channel-like structures and void spaces in the cells that enhance and facilitate the spread of oncolytic HSV (Nagano et al., 2008).

### 6.2 Off-Targeting

Although virus therapy has various benefits in controlling cancer, it has been shown to have little effect in the clinic after direct administration of HSV-1 (T-VEC) in people with melanoma due to tropism and inadequate transmission of the virus to cancer cells (Kloos et al., 2015a; Andtbacka et al., 2015). Therefore, surface alterations in oncoviruses can alleviate this problem to some extent (Jhawar et al., 2017). In tumor models, it has been revealed that insertion of a tripeptide Arg-Gly-Asp (RGD) motif in the HI loop of the adenovirus fiber knob domain can significantly enhance infection efficiency and cytotoxic effect via autophagy inhibition and apoptosis promotion (Xu et al., 2017). Another approach for targeting oncolytic adenovirus is to use different serotypes. In this regard, it has been revealed that HAdV-G52 is able to bind to polysialic acid on tumor cells, and due to the overexpression of polysialic acid on the surface of these cells, the use of HAdV-G52 can infect a variety of cancer cells. However, modifications seem to be potentially necessary to prevent neurotropism (Figarella-Branger et al., 1990; Lantuejoul et al., 1998; Tanaka et al., 2000; Suzuki et al., 2005). Other tactics for redirecting adenoviruses and targeting tumor cells by oncolytic viruses include the use of bispecific adapters capable of binding to viruses and tumor cells as well as antibody-based targeting of tumor cells by antibody single-chain variable fragments (scFvs) (Nakano et al., 2005; Belousova et al., 2008; Poulin et al., 2010; Baek et al., 2011; Kloos et al., 2015b; Bhatia et al., 2016).

### 6.3 Immune Responses

Evidence suggests that pre-existing immunity due to previous infection or immunization and shortening the virus half-life is one of the major challenges in cancer therapy with oncolytic viruses. To solve this problem, researchers mask the virus with different materials such as polymers, which can lead to virus protection, increase the virus half-life, and improve virotherapy’s effectiveness (Carlisle et al., 2013). However, due to the non-genetic nature of these changes, progeny virions cannot have these characteristics and be protected. Neutralizing antibodies are another problem in virotherapy, which can be solved by using cellular carriers as delivery vehicles (Roy and Bell, 2013). Other immune system antiviral responses, such as interferons (IFNs), can inhibit the infection via delaying virus replication. To address this problem, the use of histone deacetylase (HDAC) inhibitors such as valproic acid to induce epigenetic modifications and suppress the expression of antiviral cytokine genes has been suggested (Otsuki et al., 2008; Cody et al., 2014). However, the use of these inhibitors can have adverse effects. For example, despite enhancing the proliferation of the oncolytic virus, valproic acid can inhibit viral DNA, reduce the recruitment of effector cells such as NK cells and macrophages into the tumor microenvironment (TME), and inhibit tumor cell apoptosis (Koks et al., 2015).

The pathways leading to RNase L production can also be activated in response to viral infection, eventually destroying cellular and viral single-stranded RNA (Liang et al., 2006). Studies showed that using RNase L inhibitors such as sunitinib, which also inhibits platelet-derived growth factor receptors (PDGF-R) and VEGF, can increase the effectiveness of oncolytic viruses in cancer treatment (Tang et al., 2020). The use of other anti-angiogenic agents such as bevacizumab (Anti-VEGF) as well as cytokine therapy with transforming growth factor-beta (TGF-β), and employment of immunosuppressive drugs such as cyclophosphamide can help increase the effectiveness of virotherapy (Fulci et al., 2006; Libertini et al., 2008; Tysome et al., 2013; Han et al., 2015).

### 6.4 Impacts of Hypoxia

Based on available knowledge, hypoxia is a feature of TME in solid tumors that occurs during tumor growth and development (Bosco et al., 2020). The effect of hypoxia can be different on oncolytic viruses. For example, hypoxic conditions in the TME can modulate the oncological power as well as replication in oncolytic viruses that are dependent on cell cycle progression (Shen and Hermiston, 2005; Shen et al., 2006). In this regard, researchers have designed an oncolytic adenovirus in which the expression of the E1A gene under the promoter’s control contains the element of hypoxia response, and this genetic manipulation can lead to increased virus replication in hypoxic conditions (Hernandez-Alcoceba et al., 2002).

In contrast, under hypoxic conditions, other oncolytic viruses, including the vaccinia virus and vesicular stomatitis virus, can increase their replication potency (Connor et al., 2004; Hiley et al., 2010). Furthermore, the HSV-1 virus has been reported to exacerbate hypoxic conditions of virus replication. This ability of HSV viruses due to their tropism to low oxygen levels or oxygen-induced free radical DNA damage enhances the replication of these viruses (Aghi et al., 2009). Hypoxia-inducible factor-1 alpha (HIF-1α) has also been expressed in hypoxia that can stimulate HSV-1 proliferation-related genes (Aghi et al., 2009; Chaurasiya et al., 2018). However, infection with some oncolytic viruses, such as the Newcastle disease virus, degrades HIF-1α under hypoxic conditions and affects the expression of its target genes (Abd-Aziz et al., 2016).

### 6.5 Lack of Adequate Biomarkers for Patients Monitoring

The lack of valuable biomarkers to confirm the response of cancer patients to oncolytic viruses is another important challenge of virus therapy. Extensive tumor fluctuations also complicate the problem due to cancer patients’ specific immune system conditions who have previously tried other anticancer therapies (Turnbull et al., 2015). Studies have revealed that high mobility group box-1 (HMGB-1) in virus therapy with oncolytic adenoviruses as well as human inhibitory receptors Ig-like transcript 2 (ILT2) in the treatment of cancer with vaccinia virus can be used as predictive, prognostic, and treatment monitoring biomarkers (Zloza et al., 2014; Liikanen et al., 2015). However, further studies are needed in this area.

## 7 Concluding Remarks

Considering the relatively satisfactory outcomes of studies in the field of treatment of solid cancers such as GC using oncolytic viruses, it seems that these viruses can be used more widely in combination therapies to increase the efficiency and effectiveness of cancer treatment. However, this therapeutic approach has several challenges, and more studies are needed. In PMGC, virotherapy can limit peritoneal metastasis and tumor metastasis to the peritoneum in various ways, such as direct oncolysis of tumor cells, as well as inhibition of mechanisms and molecules involved in angiogenesis. On the other hand, inserting genes with antitumor function in the genome of oncolytic viruses for expression in virus-infected tumor cells can enhance the therapeutic effect. Viruses seem to have a wide range of unknown functions, and due to their extraordinary capabilities, such as their ability to replicate in hypoxic conditions, which is one of the drawbacks of cancer therapy, in the near future, they can be used to treat cancers to the maximum benefited performance.
